# Measuring autonomy support in special needs teachers from a self-determination theory perspective: validation of the Italian version of the learning climate questionnaire

**DOI:** 10.3389/fpsyg.2023.1183205

**Published:** 2023-07-18

**Authors:** Domenico Monacis, Francesco Sulla, Guendalina Peconio, Pierpaolo Limone

**Affiliations:** ^1^Learning Science Hub, Department of Humanities, University of Foggia, Foggia, Italy; ^2^Department of Humanities, Pegaso University, Naples, Italy

**Keywords:** special needs teachers, learning climate questionnaire, validation study, teacher-student relationship, teacher autonomy support

## Abstract

**Introduction:**

Self-determination construct is a motivation theory used in professional and educational context to foster special needs teachers’ development of metacognition, and psychological wellbeing. The Learning Climate Questionnaire (LCQ) is a validate questionnaire used to underly teachers’ professional and personal competence, and improving social, emotional and career outcomes. The present paper aims to evaluate the degree of reliability (R1) and the adherence of construct validity to the construct of self-determination (R2) of the Italian adaptation of the LCQ.

**Methods:**

A confirmatory factorial analysis was conducted to evaluate the factorial structure of the LCQ in a sample of Italian special needs teachers (*N* = 953). Teachers was asked to complete an online version of the LCQ. Construct validity was conducted by relating the learning climate with the basic psychological needs satisfaction, measured with PBNSF, and with academic motivation scale, measured with AMS.

**Results:**

The analysis showed a good reliability (R1) and construct validity of the Italian adaptation of the questionnaire, with a high internal consistency compared to those obtained in other studies (R2).

**Discussion:**

Teachers’ autonomy support and teacher-student relation can positively impact the students’ psychological factors and enhance students’ learning motivation and academic achievement. Findings reveal that higher levels of learning climate could also be a key factor in reducing teachers’ negative stress and mental health consequences.

**Conclusion:**

This study may facilitate further research about the autonomy-supportive learning climate in educational settings in Italy.

## Introduction

The new special needs teachers’ training in Italy (Ianes et al., 2020; Toto and Limone, 2021) has opened new scenarios of psychological, pedagogical, and methodological reflection aimed at enhancing inclinations, skills, potential and needs of each student in a lifelong learning perspective (Ryökkynen and Räty, 2022; Shank and Santiague, 2022; McKenzie et al., 2023). Promoting, strengthening, and extending the accessibility of opportunities for learning to special populations are essential prerogatives and development aims for ensuring children and adolescents’ inclusion in the school setting (Van Mieghem et al., 2020).

In the field of educational research, the teacher-student relationship has assumed a significant value as indicator of the quality of the didactic and learning process (Han, 2021; Pérez-Salas et al., 2021; Sulla and Rollo, 2023). Pupils’ interaction in the classroom context with teachers and peers are crucial for children’s social, behavioral, and academic development (Chen et al., 2020).

Self-Determination Theory (SDT) aims to explain the motivation that drives certain behaviors promoting a better quality of the educational process, articulated in several mini-theories: regulation types, goals, psychological needs, and autonomy supportive behaviors (Ryan and Deci, 2017, 2020; Guay, 2022).

The motivation at school may derive from reasons that differ in terms of self-determination through behaviors that presuppose intrinsic (an activity providing personal pleasure and satisfaction) or extrinsic motivation (an activity performed for instrumental reasons), respectively (Black and Deci, 2000; Ryan and Deci, 2000). Furthermore, authors have theorized several types of extrinsic motivation according to the greater or lesser degree of autonomy, as follows: identified and integrated regulation define autonomous behaviors, while external and introjected regulation characterize controlled reasons (Black and Deci, 2000; Ryan and Deci, 2000).

As for motivation, personal goals can also be classified into two broad categories: autonomous goals, such as self-acceptance, community, close relationships, and personal growth, and controlled goals, such as financial success, appearance, and popularity/fame (Deci and Ryan, 2000).

According to the SDT, humans are viewed as proactive, and humans’ motivated behaviors are guided by the Basic Psychological Needs (BPNs) for autonomy, competence, and relatedness, defined as follows: the need for autonomy is defined as the necessity to experience a sense of choice, will and personal volition; the need of competence refers to the desire to have an effective interaction with the environment (i.e., motivating challenges that stimulates personal skills); and the need of relatedness is the necessity to establish meaningful and secure emotional ties with others and peers feeling part of collectives (Ryan and Deci, 2017).

The BPNs are fundamental aspects of teacher-students’ interactions, allowing students to growth, learn, experiment and promote personal wellbeing. Whether or not BPNs are satisfied (or frustrated), they may affect student’s motivation that arise from the interaction between the person and the school environment (Guay, 2022); Gilbert et al., 2022).

Autonomy-supportive behaviors include behaviors in which someone takes others’ perspective into account promoting the best behavioral practice and guidelines (Deci and Ryan, 2000; Han, 2021). In this sense, teachers play a key role in fostering autonomous motivation in students and educational practice (Reeve and Cheon, 2021; Cho et al., 2022).

According to the SDT, intrinsic motivation would seem to be a determining factor in ensuring educational success and academic achievement (Guay, 2022); Liu et al., 2022). In particular, according to Guay (2022) both autonomous extrinsic motivation (identified and integrated regulation) and intrinsic motivation lead to better students’ academic achievement, and the satisfaction of BPNs in terms of competence, autonomy and relatedness are positively related with autonomous motivation, suggesting that autonomy-supportive teachers are important catalysts for needs’ satisfaction.

The SDT has been widely applied to a variety of contexts and domains (education, sport and physical activity, religion, health and medicine, psychotherapy, marketing science, etc.) underlying how autonomy and supportive environments can impact human behavior, functioning and wellness (Kalajas-Tilga et al., 2020; Chiu, 2022; Hardy et al., 2022; Neufeld and Malin, 2022). In the field of university enrollment, for example, affective behavior determined by attitude toward chemistry was seen to be the most important factor impacting adolescents’ future intention to enroll in chemistry university course, more than autonomy, perceived competence and attitude toward laboratory activities (Ong et al., 2022). Moreover, findings suggest that an optimal balancing between the promotion of autonomy support and educators’ clinical supervision can improve the quality of medical education courses (Sawatsky et al., 2022).

A review of the literature has also investigated the role of SDT in marketing science (Gilal et al., 2019) identifying several clusters of research in which SDT appears to be more promising in addressing marketing problems.

SDT has been also used in the field of physical education (PE) and sport. The adoption of teaching strategies by PE teacher (i.e., competitive motor tasks, the variability of activities, individual and/or team challenges, etc.) was found to positively impact students’ perception of variety, novelty, choose and praise based on effort and enhance autonomous motivation, as well as a positive learning climate and positive student–student relation are associated with better competence satisfaction and affective outcomes (White et al., 2021). Moreover, by meeting students’ basic psychological needs in the classroom, teacher can create an enjoyable setting to intrinsically motivate students to participate in learning activities, providing positive experience that empower foreign language learning (Dincer et al., 2019). The SDT’s constructs are also applied to the digital technologies’ research field in education (Chen and Zhao, 2022; Rosli et al., 2022).

In a recent systematic review Salikhova et al. (2020) highlight the significant contribution of SDT for the promotion of digital learning and students’ motivation in following e-learning courses, the prediction of students’ academic achievement and the enhancement of teachers’ motivation in the use of digital devices. Moreover, Chiu et al. (2022) explained how autonomy and perceived competence were the most important factors for developing digital literacy in grade 10 students.

Furthermore, a recent systematic review has analyzed the association of BPNs on motivation and well-being in students aged 6–14 yrs., showing a positive association between autonomous motivation and engagement and all BPNs satisfaction, and stressing teachers’ role in supporting students’ psychological needs (Conesa et al., 2022). This is particularly important for children and adolescents with special educational needs, who need everyday activities and social relationships to be adapted to their abilities and skills (Van Mieghem et al., 2020). Findings reveal that a positive classroom climate can lead to a reduction of students perceived social exclusion, and to the improvement in social skills and behavior (Beld et al., 2019; Molinari and Grazia, 2022). Furthermore, teachers’ perception of the learning climate represents a strong predictor not only of their job satisfaction and work-related emotions but is also associated with higher satisfaction and better emotions (Limone et al., 2021; Toto and Limone, 2021; Otrębski, 2022). Literature review has showed a significant positive relationship between students-teacher interaction and perceived stress and an inverse relationship of teachers’ work experience and motivation with perceived stress during didactic activities (Adigun et al., 2021; Jeon et al., 2022). Indeed, according to (Adigun et al., 2021) the unfavorable working environment and the lack of motivation represented the main sources of stress. These findings highlight that greater attention to special needs teachers’ job-related stress and well-being is needed to enhance the quality children and adolescents’ education and care, suggesting the benefits of preventive intervention programs for teachers targeting mindfulness and resilience to reduce anxiety (Ragni et al., 2023).

## The current study

Self-determination theory has been seen to foster special education teachers’ development of metacognition, motivation and strategic action, underlying professional and personal competence, and improving social, emotional and career outcomes (Black and Deci, 2000). Within this theoretical framework, Williams and Deci (1996) have developed the Learning Climate Questionnaire (LCQ), which is a 15-item questionnaire (measured on a Likert Scale) pertain to the supportive-autonomy of teacher, preceptor, or professor. The questionnaire is structured on a single underlying factor with high internal consistency (Williams and Deci, 1996), and it has never been validated in special needs teachers’ samples.

A valid and reliable questionnaire assessing special needs teachers’ supportive autonomy is necessary to orient teachers’ training and research paths in the identification of the best teaching praxis aimed at improving students’ perception of the autonomy support of their teachers; this could promote students’ autonomous self-regulation, perceived competence, and interest/enjoyment, and decrease anxiety and stress in both teachers and students.

This study used a confirmatory factorial analysis to evaluate the factorial structure of LCQ in a sample of Italian special needs teachers, with the following research questions:

R1: Does the LCQ scale translated into Italian have a degree of reliability so as to be compared to previous studies?

R2: Does the analysis of construct validity remain adherent also in adaptation to the theoretical latent construct designed to measure self-determination in the class climate?

## Participants

A total of 953 Italian special needs teachers (Table 1) were enrolled in this study between December 2022 and February 2023. While attending an in-person teacher training course at the University of Foggia, they completed a digitalized version of the self-report questionnaires included in this study. The participants signed informed consent, and they were assured of voluntary participation and anonymity. The teachers came from different Italian regions. The average age is 40 years and 82% of the surveyed population is female. Participants worked in kindergartens (20.8%), primary schools (21.5%), middle schools (27.3%), and high schools (30.4%).Considering that this study population was a convenience sample, it may not be taken as representative of the entire population of Italian special needs teachers. This study was accepted by the Ethics Committee of the University of Foggia, Italy and conducted in line with the Declaration of Helsinki.

**Table 1:** Descriptive statistics of the participants’ demographic data.

Variables	M(SD)	*N*	%	Min.	Max
Age	40.08 (7.82)			21	61
Gender					
F		786	82.5		
M		167	17.50		
Working school level					
Kindergarten		198	20.8		
Primary		205	21.5		
Middle		260	27.3		
High		290	30.4		

## Measures

Participants completed the following questionnaires:

1. The learning climate questionnaire (LCQ) was adapted by Williams and Deci (1996) from the health care climate questionnaire (Williams et al., 1996). It is typically used in specific learning settings; thus, the items are sometimes adapted to the situation being studied. If, however, it is utilized to assess a general learning climate in which each student has several teachers, the questions are stated with respect to the autonomy support of the faculty members in general. The LCQ has a long form containing 15 items and a short form containing six items. An example of item is “My instructor conveyed confidence in my ability to do well in the course.” Items are measured on a 7-points Likert scale that goes from 0 (strongly disagree) to 7 (strongly agree).
2. The basic psychological need satisfaction and frustration scale (PBNSF; Chen et al., 2015) assesses the satisfaction and frustration of the basic psychological needs defined by the self-determination theory (SDT). The Italian version of the PBNSF (Liga et al., 2018) contains 24 items (six categories with four items each) that assess both satisfaction and frustration with three psychological needs in one’s life: autonomy satisfaction (four items; e.g., “*I feel a sense of choice and freedom in the things I undertake*”), competence satisfaction (four items; e.g., “*I am confident that I can do things well*”), relationship satisfaction (four items; e.g., “*I feel that the people I care about also take care of me*”), autonomy frustration (four items; e.g., “*I feel compelled to do many things that I would not choose to do*”), competence frustration (four items; e.g., “*I have serious doubts whether I can do things well*”), and relationship frustration (four items; e.g., “*I feel that people who are important to me are cold and distant toward me*.”). Items are rated on a 5-point Likert scale ranging from 1 (strongly disagree) to 5 (strongly agree). In this study, we have selected the top eight items that evaluate autonomy, specifically, satisfaction autonomy (media items: 1, 2, 3, 4) and autonomy frustration (media items: 5, 6, 7, 8).
3. The academic motivation scale (AMS; Vallerand et al., 1992, 1993) in the Italian version by Alivernini and Lucidi (2008) consists of five subscales that assess amotivation, external regulation, introjected regulation, identified regulation, and intrinsic motivation. Each scale includes four items that are possible answers to the question “Why are you going to high school?” (e.g., “In order to obtain a more prestigious job later on”; external regulation). These answers are ranked on a 7-point Likert scale from 0 (does not match at all) to 7 (matches exactly).

## Data analysis plan

First of all, an item analysis was performed using SPSS 27 (Corp, 2020), investigating the items’ psychometric characteristics in terms of mean, standard deviation, skewness, and kurtosis. In addition to this, the Mahalanobis distance (*p* < 0.001) was calculated for all scores to identify and skip any multivariate outliers. For validation, preliminary tests were conducted relating to reliability (R1), specifically test–retest and internal consistency between the variables of a scale, with Cronbach’s alpha (> 0.700) reported in the next section. Construct validity (R2) was measured by confirmatory factor analysis based on the previous four studies (Williams et al., 1994; Williams and Deci, 1996; Williams et al., 1997; Black and Deci, 2000). This process was conducted through the evaluation of the model fit and of the convergent and discriminant validity. The reliability of the construct was also evaluated with ICR and Cronbach’s alpha. Finally, the discriminant validity of all constructs was calculated by evaluating the quadric values.

## Results

Descriptive statistics related to the average scores in the the subscales and total scores scored by participants in the scales utilized for the current investigation are reported in Table 2. As regards the LCQ, higher average scores indicate a higher level of perceived autonomy support. In this sample a medium score of perceived autonomy support was found. With respect to the Autonomy subscales of the PBNSF, a medium-low score of autonomy (i.e., perceived psychological freedom in carrying out an activity) was found, with autonomy frustration being higher that autonomy satisfaction in this sample. As regards the AMS, the highest average score was found in Identified regulation, while the lowest was found in amotivation. Moreover, the Relative Autonomy Index (RAI) was calculated. The RAI measure is an indicator of a person’s overall motivational orientation. Positive scores represent more autonomous regulation; negative scores represent more controlling regulation. RAI is positively correlated with intensions to persist in school (Vallerand et al., 1993). The participants of this study reported an average high and positive score, that indicated a more autonomous regulation.

**Table 2:** Descriptive statistics of average scores in the subscales and total scores of the utilized tools.

		*N*	Min.	Max.	Mean	SD	Skewness		Kurtosis	
								Std. Error		Std. Error
	LCQ	953	1.00	7.00	2.556	1.25.397	1.040	0.079	0.636	0.158
PBNSF	Autonomy Satifaction	953	1.00	5.00	1.864	85.275	1.313	0.079	1.692	0.158
	Autonomy Frustration	953	1.00	5.00	3.659	1.06272	-0.573	0.079	-0.436	0.158
	Autonomy	953	1.00	5.00	2.102	79.836	0.673	0.079	0.120	0.158
AMS	Amotivation	953	1,00	4.00	1.294	59.056	2.251	0.079	4.506	0.158
	External Regulation	953	1.00	4.00	1.644	72.864	1.083	0.079	0.350	0.158
	Introjected regulation	953	1.00	4.00	1.739	63.718	0.993	0.079	0.756	0.158
	Identified regulation	953	1.00	4.00	3.597	56.374	-1.632	0.079	2.653	0.158
	Intrinsic regulation	953	1.00	4.00	3.552	57.107	-1.520	0.079	2.394	0.158
	Relative Autonomy Index	953	−6.50	9.00	6.468	276.000	−1.467	0.079	1.649	0.158

Regarding the validation of the LCQ, CFA was conducted using SPSS AMOS software, which uses the maximum likelihood (ML) algorithm to estimate the results. After defining the model in the software and executing the analysis, four main phases were conducted to examine construct validity: (1) assessment of model fit, (2) assessment of convergent validity, (3) assessment of discriminant validity, and (4) respecification of the model (if necessary).

The first step was to execute a reliability analysis to check if an acceptable level of reliability was present to proceed to the assessment of validity (R1). Reliability was assessed using Cronbach’s alpha (α). The table below (Table 3) shows the descriptive statistics and alpha coefficients for the scales under study. All three coefficients were above 0.700, which means the scales have good reliability. Item 13 was reversed but still showed poor item-total correlation (0.191); it might indicate that this item does not measure the same aspect as the whole scale. Since the total scale showed good reliability, the item was kept for the validity analysis phase (see next section).

**Table 3:** Descriptive statistics and alpha coefficients.

Construct	Item	Mean	Standard deviation	Item-total correlation	**α**
Learning climate	LCQ_1	2.501	1.519	0.813	0.961
	LCQ_2	2.483	1.526	0.836	
	LCQ_3	2.372	1.499	0.786	
	LCQ_4	2.471	1.561	0.834	
	LCQ_5	2.309	1.522	0.866	
	LCQ_6	2.655	1.602	0.818	
	LCQ_7	2.359	1.499	0.799	
	LCQ_8	2.424	1.538	0.877	
	LCQ_9	2.241	1.452	0.843	
	LCQ_10	2.490	1.474	0.826	
	LCQ_11	2.510	1.522	0.847	
	LCQ_12	2.805	1.625	0.808	
	LCQ_13_Reversed	2.863	2.060	0.191	
	LCQ_14	2.799	1.528	0.746	
	LCQ_15	2.939	1.643	0.706	
Autonomy satisfaction	PBNSF_1	1.878	0.950	0.672	0.886
	PBNSF_2	1.969	0.996	0.803	
	PBNSF_3	1.901	0.984	0.787	
	PBNSF_4	1.733	0.994	0.748	
Autonomy satisfaction	PBNSF_5	3.663	1.247	0.629	0.854
	PBNSF_6	3.733	1.220	0.741	
	PBNSF_7	3.527	1.329	0.678	
	PBNSF_8	3.682	1.280	0.733	
Amotivation	AM_5	1.255	0.674	0.707	0.853
	AM_10	1.346	0.745	0.706	
	AM_13	1.243	0.656	0.705	
	AM_18	1.372	0.756	0.654	
External regulation	AM_1	1.593	0.929	0.568	0.801
	AM_8	1.919	0.983	0.683	
	AM_15	1.592	0.914	0.508	
	AM_20	1.539	0.892	0.708	
Introjected regulation	AM_6	1.760	1.059	0.508	0.694
	AM_11	2.591	1.083	0.316	
	AM_14	1.346	0.741	0.542	
	AM_19	1.272	0.671	0.492	
Identified regulation	AM_3	3.669	0.631	0.652	0.830
	AM_9	3.524	0.746	0.612	
	AM_12	3.586	0.702	0.695	
	AM_17	3.548	0.715	0.671	
Intrinsic regulation	AM_2	3.661	0.634	0.636	0.805
	AM_4	3.534	0.699	0.655	
	AM_7	3.597	0.715	0.638	
	AM_16	3.327	0.857	0.534	

The statistics that were used to assess model fit and their rules of thumb are presented in Table 4.

**Table 4:** The below data identify the statistics used to assess the model fit and the rules of thumb.

Fit index	Rules of thumb
Root mean square error of approximation (RMSEA)	RMSEA <0.08
Comparative fit index (CFI)	CFI > 0.90
Normed fit index (NFI)	NFI > 0.85

After the assessment of model fit, convergent and discriminant validity were examined. Analyzing the Convergent Validity and Reliability shows that the model (with all elements) showed an acceptable fit (χ^2^ (832) = 3544.44; *p* < 0.001; χ^2^/df = 4.260; RMSEA = 0.053; CFI = 0.919; NFI = 0.897). A second model (without both items) showed an even better fit (χ^2^ (751) = 3124.18; p < 0.001; χ^2^/df = 4.160; RMSEA = 0.053; CFI = 0.928; NFI = 0.907).

The main objective of testing a measurement model is to test construct validity (R2). Construct reliability was tested using the composite reliability index (which is based on factor loadings) and Cronbach’s alpha (which is based on correlations). Table 5 provides a summary of the indicators used to measure the construct validity and reliability.

**Table 5:** This table identifies and summarizes the indicators used to measure construct validity and reliability.

	Definition	Rules of thumb
Indicator of convergent validity		
Factor loadings (λ)	Correlation between the original variables and the factors, and the key to understanding the nature of a particular factor. Squared factor loadings indicate what percentage of the variance in an original variable is explained by a factor.	In the case of high convergent validity, high one-factor loadings would indicate that they converge on a common point, the latent construct. At a minimum, all factor loadings must be statistically significant. Because a significant load can still have quite weak strength, a good rule of thumb is that standardized loading estimates should be 0.5 or higher and ideally 0.7 or higher.
Average Variance Extracted (AVE)	A summary measure of convergence among a set of items representing a latent construct. It is the average percentage of variation explained (variance extracted) among the items of a construct.	An AVE of 0.5 or higher suggests adequate convergence. An AVE of less than 0.5 indicates that, on average, more error remains in the items than is explained by variance by the latent factor structure imposed on the measure.
Indicator of discriminant validity		
AVE and correlations (*p*)	The squared variance extracted estimates for a construct should be greater than the correlation estimates between this and other constructs.	Squared AVE > *p* Fornell & Larcker (1981).
Indicator of discriminant validity		
Construct Reliability (CR)	A measure of reliability and internal consistency of the measured variables representing a latent construct. Must be established before construct validity can be assessed. It is computed from the squared sum of factor loadings for each construct and the sum of the error variance terms for a construct.	A value of 0.7 or higher suggests good reliability. Reliability between 0.6 and 0.7 may be acceptable, provided that other indicators of a model’s construct validity are good.
Cronbach’s Alpha	Cronbach’s alpha is a coefficient that represents the proportion of total variance among items that is due to the construct that it intends to measure.	A value of 0.7 is the minimum acceptable Pallant (2020).

The model (with all items) showed acceptable fit (χ^2^ (832) = 3544.44; *p* < 0.001; χ^2^/df = 4.260; RMSEA = 0.053; CFI = 0.919; NFI = 0.897). Convergent validity was not achieved for the construct ‘Introjected Regulation’ (AVE = 0.395). When examining individual factor loadings, item ‘AM_11’ showed poor loading (λ = 0.325) and was therefore deleted before running the analysis a second time. An additional item showed a factor loading lower than 0.500 (‘LCQ_13_Rescored’) and was also excluded from the ‘Learning Climate’ scale.

A second model (without both items) showed an even better fit (χ^2^ (751) = 3124.18; p < 0.001; χ^2^/df = 4.160; RMSEA = 0.053; CFI = 0.928; NFI = 0.907). The ‘Introjected Regulation’ scale still showed an AVE lower than what would be ideal to reflect convergent validity but barely below the acceptable threshold (AVE = 0.492). Since factor loadings of the three remaining items in the scale were all above 0.500, which is also evidence of convergent validity, the authors decided to proceed with this solution without excluding any additional item. The final results of convergent validity and reliability are shown in Table 6.

**Table 6:** Convergent validity and reliability.

Item		Construct	Loadings	AVE	CR	Cronbach’s alpha
LCQ_1	<−--	Learning_climate	0.833	0.696	0.970	0.969
LCQ_2	<−--		0.854			
LCQ_3	<−--		0.803			
LCQ_4	<−--		0.854			
LCQ_5	<−--		0.883			
LCQ_6	<−--		0.842			
LCQ_7	<−--		0.819			
LCQ_8	<−--		0.896			
LCQ_9	<−--		0.861			
LCQ_10	<−--		0.840			
LCQ_11	<−--		0.864			
LCQ_12	<−--		0.825			
LCQ_14	<−--		0.763			
LCQ_15	<−--		0.726			
PBNSF_1	<−--	Autonomy_Satisfaction	0.724	0.667	0.889	0.886
PBNSF_2	<−--		0.858			
PBNSF_3	<−--		0.853			
PBNSF_4	<−--		0.825			
PBNSF_5	<−--	Autonomy_Frustration	0.706	0.597	0.855	0.854
PBNSF_6	<−--		0.822			
PBNSF_7	<−--		0.751			
PBNSF_8	<−--		0.806			
AM_18	<−--	Amotivation	0.736	0.593	0.854	0.853
AM_13	<−--		0.785			
AM_10	<−--		0.776			
AM_5	<−--		0.783			
AM_20	<−--	External_regulation	0.831	0.520	0.810	0.801
AM_15	<−--		0.615			
AM_8	<−--		0.768			
AM_1	<−--		0.648			
AM_19	<−--	Introjected_regulation	0.779	0.492	0.740	0.700
AM_14	<−--		0.752			
AM_6	<−--		0.551			
AM_17	<−--	Identified_regulation	0.768	0.550	0.830	0.830
AM_12	<−--		0.743			
AM_9	<−--		0.676			
AM_3	<−--		0.776			
AM_16	<−--	Intrinsic_regulation	0.687	0.518	0.866	0.805
AM_7	<−--		0.719			
AM_4	<−--		0.726			
AM_2	<−--		0.730			

After determining convergent validity, the discriminant validity was assessed. Table 7 shows the squared AVE values (diagonal) along with correlations among constructs obtained through CFA (non-diagonal values). All constructs showed good discriminant validity since the squared AVE (diagonal) values are all higher than the correlations between the constructs. The only exception was between identified and intrinsic regulation, which showed a very high correlation (r = 0.950), higher than the squared AVE of both constructs. Therefore, a researcher should be mindful when using both constructs on any predictive algorithm (i.e., regression models) since they could lead to multicollinearity. If this high correlation is theoretically plausible, then the researcher may proceed with both constructs without a problem.

**Table 7:** Discriminant validity.

	Learning climate	Autonomy satisfaction	Autonomy frustration	Amotivation	External regulation	Introjected regulation	Identified regulation	Intrinsic regulation
Learning climate	0.834							
Autonomy satisfaction	0.588	0.817						
Autonomy frustration	−0.260	−0.444	0.773					
Amotivation	0.350	0.428	−0.393	0.770				
External regulation	0.246	0.319	−0.300	0.540	0.721			
Introjected regulation	0.179	0.271	−0.273	0.713	0.718	0.701		
Identified regulation	−0.348	−0.471	0.378	−0.593	−0.176	−0.278	0.742	
Intrinsic regulation	−0.342	−0.413	0.363	−0.557	−0.204	−0.204	0.950	0.719

## Discussion

The primary main aim of the current study (R1) was to evaluate the degree of reliability and validity of the Italian adaptation of the LCQ (Williams and Deci, 1996), and to assess the adherence of the construct validity to the theoretical latent aim for self-determination measure in the class (R2) in a sample of special education teachers.

To the best of our knowledge, the present study presented a first contribution to the Italian validation of the learning climate questionnaire (Williams and Deci, 1996) in a sample of Italian special needs teachers. Previous studies investigated and evaluated the construct validity of the questionnaire in a sample of chemistry (Black and Deci, 2000), internal medicine and surgery (Williams et al., 1994, 1997), medical students (Williams and Deci, 1996).

Overall, the reliability analysis showed a good reliability, model fit and construct validity of the Italian version of the questionnaire. The Learning Climate dimension showed a high internal consistency (R2; α > 0.700) compared to those obtained in other validation studies (Williams et al., 1994, 1997; Williams and Deci, 1996; Black and Deci, 2000). Moreover, the discriminant validity analysis shows that there is good discriminant validity, as the square AVE values were higher than the correlations between the constructs.

Results of construct validity analysis was similar to other validation studies (Núñez et al., 2012; Yu et al., 2018; Bean et al., 2020).

The correlation coefficients are almost all in the expected direction, with some exceptions. Autonomy frustration was negatively correlated to LCQ scores. People that perceived higher autonomy support are also feeling less autonomy frustration. This means that perceived autonomy support may represents a protective factor. This is also true for intrinsic motivation in attending the course to become qualified special needs teachers.

Indeed, regarding to the academic motivation, amotivation scores were found to be fairly low in this sample. In contrast, highest scores were found in identified and intrinsic regulations, that can be considered as the most self-determined form of behaviors. These may represent protective factors in completing the in-service training course.

The values of the average scores of the subscales (Tab. 2) reveal innovative interpretation of the data relating to the dimensions investigated by the three scales (LCQ, AMS, and PBNSF). Based on the RAI calculation of the AMS, it appears that with positive scores there is a more autonomous regulation, while with negative scores there is a controlling regulation. Furthermore, from the subscales (AMS), it is possible to evaluate intrinsic or extrinsic motivation. The LCQ scale shows that the higher the score, the greater the support for perceived autonomy.

The results of the present study can be useful to define some methodological implications and application in educational contexts. Indeed, based on previous research studies, the following list of teacher’s behaviors that support students’ autonomy can be pointed out: present and explain the learning contents clearly, promote student’s self-perception through autonomy language, leave the possibility for students to approach the content of learning in a personal way, encouraging autonomy decision and free choice to promote intrinsic motivation (Núñez and León, 2016; Zhao and Qin, 2021). Findings reveal that the positive association between teacher’s autonomy support and students’ school engagement is mediated by students’ perception of self-competence (Li et al., 2020), as well as perceived self-efficacy and school engagement mediate the effect of autonomy support on academic achievement (Gutiérrez and Tomás, 2019).

According to recent literature findings, in fact, teachers’ autonomy support and teacher-student relation positively impact the students’ psychological factors (Simon and Salanga, 2021), reduce depression in primary and middle school children (Zhang et al., 2022), promote more adaptive learning behavior (Schweder and Raufelder, 2022), and enhance students’ learning motivation and academic achievement (Admiraal et al., 2022).

Thus, we may hypothesize that self-compassion could also be a key factor in reducing teachers’ negative stress and mental health consequences.

## Limitations and future research

Participants in the present study are students who participated in a special needs teachers training course. However, there are two main limitations due to the type of sampling and a gender bias. According to participants’ recruitment, a convenience sampling was applied. Despite the advantages regarding the easier and more practical accessibility of the participants, there are also some disadvantages typical of the convenience sampling, such as usual sampling bias, sample not representative of the population, and the impossibility to assess the degree of sampling bias. Due to these limitations, data cannot be generalized to a larger population.

Future studies should consider other type of sampling (i.e., systematic, stratified or cluster sampling) to improve generalizability of the results. Moreover, due to the larger number of females compared to males participants, the invariance test was not performed to assess differences or functional equivalence of the LCQ scale across gender groups. Future studies may be conducted on larger and more balanced samples.

## Conclusion

In addition to statistical and theoretical issues, the use of the LCQ may have significant implications for scientific purposes and special education needs teachers’ training as well. Indeed, the LCQ scale resulted to be useful in investigating how the quality of teacher-students relationship impacts psychological and academic outcomes and contribute to improve classroom climate in every school grade and different educational settings (e.g., Brandisauskiene et al., 2021; Simon and Salanga, 2021).

The current investigation has some limitations. The participants in the current study were only those who participated in an in-service teacher training course at University of Foggia – in the South of Italy – to become special needs teachers, and this suggests caution regarding the generalizability of results.

The LCQ was utilized to assess the general learning climate in a setting where each trainee has several instructors, and each instructor meets trainees for a limited period of time (i.e., 30 h in a year). Moreover, classes were generally attended by more than 100 trainees, and this might have an influence on perceived autonomy support of the trainees, as well as on their autonomy. Another important variable that should be considered is that, although almost all participants had a job activitiy, the mandatory nature of lessons and activities during the training course (lessons, internships in schools, laboratories on Information and Communication Technologies) might have affected the results of autonomy frustration and autonomy satisfaction.

Moreover, most of the participants were female. However, because of this it was not possible to analyze the scale invariance in terms of gender. Future studies should investigate the gender factor by using more homogeneous samples. Another limit is the cross-sectional design of the study. Future investigations should collect longitudinal data to provide support for the validity of the LCQ.

Despite these limitations, the current study has met its aim to examine psychometric properties of the LCQ which is one of the most commonly used measures for perceived autonomy support. This study may facilitate further explorations into the measurement of an autonomy-supportive learning climate in educational settings in Italy.

## Data availability statement

The raw data supporting the conclusions of this article will be made available by the authors, without undue reservation.

## Ethics statement

The studies involving human participants were reviewed and approved by University of Foggia. The patients/participants provided their written informed consent to participate in this study.

## Author contributions

DM, FS, GP designed and carried out the study. GP collected the data. FS performed statistical analysis. DM, FS, GP contributed to the analysis of the results and to the writing of the manuscript. PL supervised the study design and the manuscript draft. All authors contributed to the article and approved the submitted version.

## Conflict of interest

The authors declare that the research was conducted in the absence of any commercial or financial relationships that could be construed as a potential conflict of interest.

## Publisher’s note

All claims expressed in this article are solely those of the authors and do not necessarily represent those of their affiliated organizations, or those of the publisher, the editors and the reviewers. Any product that may be evaluated in this article, or claim that may be made by its manufacturer, is not guaranteed or endorsed by the publisher.
